# Fetal functional connectivity: Examining the role of prenatal maternal depression symptoms using graph theory

**DOI:** 10.1016/j.dcn.2025.101585

**Published:** 2025-06-16

**Authors:** Ellyn Reed, Lanxin Ji, Marjorie Beeghly, Amyn Majbri, Tanya Bhatia, Mark Duffy, Iris Menu, Christopher Trentacosta, Moriah E. Thomason

**Affiliations:** a Department of Psychology, Wayne State University, Detroit, MI, United States; b Merrill Palmer Skillman Institute for Child & Family Development, Wayne State University, Detroit, MI, United States; c Department of Child and Adolescent Psychiatry, New York University Medical Center, New York, NY, United States; d Université Paris Cité, LaPsyDÉ, CNRS, Paris F-75005, France

**Keywords:** Graph theory, Fetal fMRI, Prenatal, Prenatal maternal depression, Fetal brain neurodevelopment

## Abstract

Altered fetal brain function is proposed as a mechanism underlying the relationship between prenatal maternal depression (PMD) and neurodevelopmental outcomes in offspring. This study investigated the association between PMD symptoms and fetal brain functional connectivity (FC) using graph theory. A total of 123 pregnant women participated in the study, completed the Center for Epidemiologic Studies Depression Scale (CES-D), and underwent fetal MRI scans. Results revealed a significant relationship between elevated PMD symptoms and reduced global efficiency in the right insular region of the fetal brain. However, because fetal age was not associated with local or global efficiency in the insular brain region, we cannot determine if the PMD-related reduction in insula global efficiency is indicative of an accelerated or delayed developmental pattern. This study is one of the few to examine fetal brain connectivity in relation to prenatal maternal depression, providing valuable insights into early neurodevelopmental risks and potential targets for early intervention.

## Highlights

• Graph theory applied to analyze fetal brain FC in the context of PMD.
• PMD linked to decreased global efficiency in fetal right insular region.
• Fetal age was not related to right insular efficiency.
• Prenatal PMD appears to have mixed effects on fetal brain network efficiency.
• PMD-related alterations in fetal brain FC show need for prenatal mental health care.

## Graphical Abstract

**Figure:**
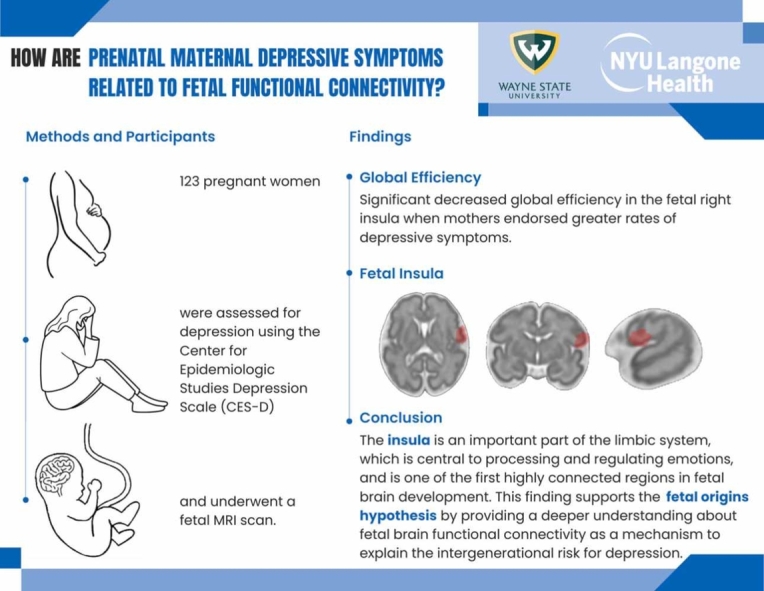

## 1 Introduction

Depression is the most common psychiatric disorder during pregnancy, affecting 10–14 % of women (Van Niel and Payne, 2020). Depression has been linked to preterm birth, low birth weight, poor infant neurodevelopment, and offspring mental health concerns (Jarde et al., 2016, Smith et al., 2020). The pathway by which prenatal maternal depression (PMD) influences child development needs to be better understood to provide effective and timely psychological intervention to populations at most risk.

One of the hypothesized mechanisms that could explain the transgenerational risk for depression and other psychiatric disorders is altered fetal brain functioning. Existing evidence that supports fetal brain programming comes from neuroimaging studies of infants born to mothers with elevated symptoms of PMD (Duan et al., 2019, El Marroun et al., 2016, Rifkin-Graboi et al., 2013, Qiu et al., 2015). Qiu et al. (2015) found that infants of mothers with prenatal depression had increased connectivity between the amygdala and several brain regions, including the insula, bilateral anterior cingulate, and ventromedial prefrontal cortex. These findings are similar to functional connectivity patterns seen in adolescents and young adults with depression (Kerestes et al., 2014, Qiu et al., 2015). Likewise, Posner et al. (2016) found atypical amygdala-prefrontal functional connectivity in infants who were exposed to maternal prenatal depression. Combined, these data suggest that PMD may alter connectivity across large-scale fetal brain networks, but direct evidence linking PMD to *fetal* brain connectivity is currently lacking.

An important approach to understanding neural mechanisms is to consider the overall network architecture, as compared to focusing on specific brain regions or systems. Indeed, studies of prenatal depression using graph theoretical analyses have demonstrated associations between PMD and network graph features in children. An analysis conducted by Donnici et al. (2023) reported that PMD was associated with altered global efficiency and node degree, which both reflect how effectively information is exchanged across networks, in children ages 2.6–8 years old. Global efficiency in the limbic system and average node degree increased with age. Children whose mothers reported higher rates of depression during the third trimester of pregnancy were observed to have diminished global efficiency and average node degree compared to what was expected for their age. Reduced child network integration between regions implicated in emotion regulation within the limbic system in children exposed to PMD, as demonstrated by Donnici et al. (2023), is consistent with prior literature in adults. Adult research has shown that reduced global efficiency in the limbic system is associated with current symptoms of depression (Korgaonkar et al., 2014, Yun and Kim, 2021). Changes *in utero* may put an individual at risk for developing depression later in life. Fetal brain topography assessed via graph-theory techniques can, therefore, contribute to the work conducted on child and adult samples, providing a better understanding of the mechanisms underlying the association between PMD and child outcomes.

Our analytic approach focuses on local and global efficiency, which are graph metrics from network analyses that are interpreted as reflecting the architecture supporting communication across brain systems. Global efficiency describes how effectively information is transferred across the entire network, serving as an indicator of overall connectedness, while local efficiency reflects how efficiently information is exchanged with immediate neighboring regions, providing a level of localized integration within a network. The association between PMD and child functional brain development has been explored using a whole-brain graph-based analysis (e.g., Cao and He, 2021), but not in a fetal sample. Using fetal neuroimaging techniques in the current study to examine the association between PMD and fetal brain is critical, as it can isolate prenatal exposure to maternal depression from postnatal factors.

The main objective of the current study was to examine associations between maternal symptoms of depression during the prenatal period and fetal functional connectivity using graph theory. We hypothesized that elevated PMD symptoms will be associated with reduced network efficiency. To construct the brain graph, we conducted spatial spectral clustering to define a collection of regions, or “nodes”, and we then interrogated connections, or “edges”, between these data-driven defined regions. We then explored the effect of PMD on global and local efficiency of the fetal brain, and how these measures relate to fetal age, given the rapid development of brain circuitry throughout pregnancy, to determine if our PMD findings are indicative of accelerated or delayed development. The current study’s sample consists primarily of low-income African American pregnant women who are at greater risk of experiencing PMD (Melville et al., 2010).

## 2 Methods

### 2.1 Datasets and procedure

The current study is part of a larger longitudinal research project conducted at Wayne State University, which examines neural, socioemotional, and behavioral development in utero and throughout childhood. Pregnant women were recruited during routine prenatal care appointments at the Hutzel Women’s Hospital in Detroit Michigan. Healthy mothers underwent a fetal MRI and completed questionnaires related to demographics and depression.

### 2.2 Participants

Participants must have met the following inclusion criteria to participate in the larger research study: English as their primary language, between 18 and 40 years of age, carrying a singleton pregnancy, and no MRI contraindications. The current analysis included a subgroup of 123 participants who completed a depression questionnaire during the prenatal research assessment and underwent a fetal MRI scan that was successfully processed according to the preprocessing quality guidelines that are detailed below (See Table 1). Mothers predominantly self-identified as African American (85.4 % African American/Black, 7.3 % White, 5.7 % Biracial, 0.8 % Asian American; see Table 2). Participants most commonly self-reported being single (66.7 %), having an annual household income of less than $10,000 (52.3 %), and having a GED or high school level of educational attainment (39.8 %; see Table 2).

**Table 1:** Participant Screening Process.

Exclusion Criteria	Number of participants removed
*Initial sample size*	212
Fewer than 105 frames of low motion fMRI data	44
Residual high motion after scrubbing	2
Gestational age at MRI < 24 weeks	3
Data file errors	2
Duplicate MRI scans per participant	11
Missing depression data	24
Missing covariate data	3
Total excluded	89
*Final sample size*	123

Participants were excluded if they had fewer than 105 frames of low motion data, if motion was significant even after removal of high motion frames (mean max xyz > 1.5 mm, or mean max pry > 1.5 degrees), and if gestational age was less than 24 weeks. In two cases, there were unspecified fMRI file errors. Only the highest quality fMRI run was retained per participant and this selection was determined by frame count and motion. Participants with missing depression or covariate data (e.g., maternal education) were excluded.

**Table 2:** Demographics.

	*n*	*%*
Fetal sex		
Male	75	61
Female	48	39
Race		
White	9	7.3
African American	105	85.4
Asian American	1	0.8
Other	1	0.8
Bi-racial	7	5.7
Marital status		
Single	78	66.7
Married/partnered	38	32.5
Divorced	1	0.9
Not Reported	6	4.9
Highest education level		
No GED/No HS diploma	21	17.1
GED/HS diploma	49	39.8
Some college	45	36.6
2 yr college degree	2	1.6
4 yr college degree	3	2.4
Masters	1	0.8
Doctorate	2	1.6
Annual Household Income		
< 10,000	58	52.3
10,000–20,000	23	20.7
20,000–30,000	14	12.6
30,000–40,000	6	5.4
40,000–50,000	1	0.9
50,000–60,000	2	1.8
80,000–100,000	1	0.9
100,000–120,000	3	2.7
120,000–140,000	1	0.9
140,000–160,000	1	0.9
220,000–250,000	1	0.9
Not Reported	12	9.8

### 2.3 Covariates

Covariates were selected *a priori* based on their associations in the literature with the primary variables of interest, fetal fMRI and PMD symptoms; covariates included fetal gestational age (GA), fetal sex, maternal age and educational attainment. Fetal GA at the time of scan (*M* = 31.27, *SD* = 3.77, *min* = 24.14, *max* = 38.71) was determined by ultrasound and biological sex was extracted from medical records. 61 % of the sample were male (*n* = 75). Maternal age and educational attainment were collected via maternal self-report. At the time of enrollment, the mother’s age ranged from 18.15 – 40.80 years old (*M* = 25.54, *SD* = 4.50; see Table 2). Follow up sensitivity analyses were conducted to isolate potential influence of maternal mental and physical health on observed effects.

### 2.4 Measures

#### 2.4.1 Prenatal maternal depression (PMD)

The Center for Epidemiologic Studies – Depression Scale (CES-D) (Radloff, 1977) was completed by the mothers during the prenatal study visit. This questionnaire measures symptoms of depression in the past two weeks and adopts a 4-point Likert scale where 0 = rarely or none of the time (less than 1 day), 1 = some or a little of the time (1–2 days), 2 = occasionally or a moderate amount of the time (3–4 days), and 3 = most or all of the time (5–7 days). The 20 items were scored from 0 to 3, with a possible total score range of 0–60, and a clinical cutoff of 16 (Henry et al., 2018, Lewinsohn et al., 1997). The questions were selected for the CES-D based on the diagnostic criteria in the Diagnostic and Statistical Manual of Mental Disorders at the time (DSM-II, APA 1968). The CES-D has adequate test-retest reliability, and validity has been established for different ethnic, racial, gender, and age groups (Nguyen et al., 2004, Radloff, 1977, Roberts, 1980). The CES-D total depression score was used in the current analysis, and in our sample had good internal reliability (α =.80).

#### 2.4.2 Fetal fMRI

Fetal fMRI was obtained using a 3 T Siemens Verio 70 cm open-bore system with a 550-g abdominal 4-channel Siemens Flex Coil (Siemens Corp). For each participant, 360 axial frames (12 min) of blood oxygen level–dependent echo-planar imaging (BOLD EPI) data were collected with either of the following scan sequence parameters: (1) 12-min single-echo fMRI: TR/TE = 2000/30 ms; resolution = 3.4 × 3.4 × 4 mm^3,^ flip angle: 80 degrees. (2) 12-min multi-echo (ME) fMRI: TR = 2000 ms; TE = 18, 34, 50 ms (3 echoes); flip angle: 83 degrees; voxel size: 3.5 × 3.5 × 3.5 mm^3^. This sequence was repeated when time permitted.

### 2.5 fMRI data preprocessing

#### 2.5.1 Preprocessing

Preprocessing was performed using a combination of FSL (https://fsl.fmrib.ox.ac.uk/fsl/fslwiki/), Statistical Parametric Mapping (SPM12) software (https://www.fil.ion.ucl.ac.uk/spm/ software/spm12/), and other image processing toolboxes. Fetal image processing began using a convolutional neural network (CNN) to automatically segment the fetal brain from surrounding maternal tissue (Rutherford et al., 2022). To correct the suboptimal performance of the CNN, we applied the motion profiles estimated from the CNN-extracted brains on a manually traced brain mask to generate a 4-dimensional brain mask moving along with the raw fMRI data. BrainSuite (http://brainsuite.org/) was used to manually edit three-dimensional masks around single reference images. All manually edited masks were quality-assured by a second reviewer. Motion was estimated with FSL FLIRT, and motion-contaminated frames (i.e., scrubbing) were removed with thresholds of framewise displacement > 1.5 mm, DVARS > 132.69, and Sørensen–Dice coefficient (DC) below 0.9 between the individual participant’s frame and the reference frame. Participants with fewer than 105 low-motion frames or a motion drift greater than 0.5 mm across the time series were excluded from further analyses (also see Table 1). The decision to use a 105-frame cutoff aims to optimize data length while also including the maximum number of subjects.

After quality-assurance, data was used to estimate T2* maps, which contain physiological information, and were then used to generate weights for computing a weighted average of the data acquired at multiple echoes. This weighted average data (i.e., the optimal combined data) took advantage of the higher signal in earlier echoes and the higher contrast at later echoes. Subsequent preprocessing steps including normalization to standard space (GA = 32 weeks), spatial smoothing with a 3-mm kernel, ICA denoising, and CompCor denoising were performed using Functional Connectivity Toolbox (CONN21.a) (Behzadi et al., 2007; Whitfield-Gabrieli and Nieto-Castanon, 2012). Specifically, during CompCor, the regression of potential confounding effects includes white matter timeseries (5 CompCor noise components), CSF timeseries (5 CompCor noise components), motion regressors and their first order derivatives (12 components), and linear trends (2 factors) within each functional run, followed by bandpass frequency filtering of the BOLD timeseries between 0.008 Hz and 0.09 Hz (Hallquist et al., 2013, Nieto-Castanon, 2020, Penny et al., 2011). For normalization, a mapping between functional native space and the template space was constructed by concatenating a linear transformation between the functional scan and the age-matched template, and a sequence of nonlinear transformations between templates of adjacent ages (e.g., 24 and 23, 25 and 24, etc.). This gradual alignment minimized the risk of gross misalignments due to differences in brain topology across GA.

#### 2.5.2 Functional connectivity matrix construction

Preprocessed fetal datasets were submitted to the SLIC toolbox (https://www.nitrc.org/projects/slic/) to generate a data-driven, group balanced functional atlas consisting of 197 functional parcels (Wang and Wang, 2016). Average time series were calculated for each parcel and used to construct the 195 × 195 functional connectivity matrices of Pearson correlation coefficients. Based on functional connectivity matrices, we estimated the graph theory measures of global efficiency (GE) and local efficiency (LE) for each subject in CONN. For each ROI, we defined global efficiency as the average inverse shortest path distance from node n to all other nodes in the graph, and local efficiency as the average efficiency across all nodes in the local subgraph of node n (the subgraph consisting only of neighboring nodes).

### 2.6 Statistical Analysis

Descriptives and measures of variability were examined for all variables. Linear regression assumptions were assessed in several ways. We examined a scatterplot, histogram, and QQ plot and determined that the assumptions of normality and linearity of residuals as well as homoscedasticity were met. The assumption of independence of errors was tested and met using the Durbin Watson test (*DW* = 1.80, *p* = .555). We detected 2 possible outliers by calculating the Mahalanobis Distance for each case, the leverage each case had on the regression model, and Cook’s distance from the regression output. The two possible outliers had a higher level of education than the majority of the sample because they both completed graduate school. We opted to retain these outliers because they are valid data points reflective of the true community sample, and all other linear regression assumptions were met.

#### 2.6.1 Effect of prenatal maternal depression on the fetal brain

The CES-D total score showed acceptable distribution normality (depression skew = 0.85). To test the hypothesis that the severity of PMD symptoms was related to fetal functional graphs, group-level analyses were performed using a General Linear Model (GLM; Nieto-Castanon, 2020). A GLM was estimated across individuals, with global efficiency or local efficiency included as dependent variables, and groups or other subject-level identifiers as independent variables. For the depression focused analysis, relevant covariates determined by theoretical associations with depression and fetal brain FC, including the fetuses’ gestational age at the time of scan, fetal sex, maternal age, and maternal educational attainment, were entered into the first step. In a second step, PMD symptoms was entered as the predictor of fetal graph measures of FC.

#### 2.6.2 Age-related change in network efficiency

The same group-level analysis detailed above was performed on the sample to examine the association between fetal age and graph metrices. It’s important to understand age related changes in our specific fetal sample to aid interpretation of the anticipated PMD and fetal FC associations, as the fetal brain is rapidly developing. Here, the covariate of fetal sex was entered into step one, then fetal age at time of scan was entered in step two as the predictor of fetal graph measures of FC

## 3 Results

### 3.1 Prenatal maternal depression

**Fig. 1:**
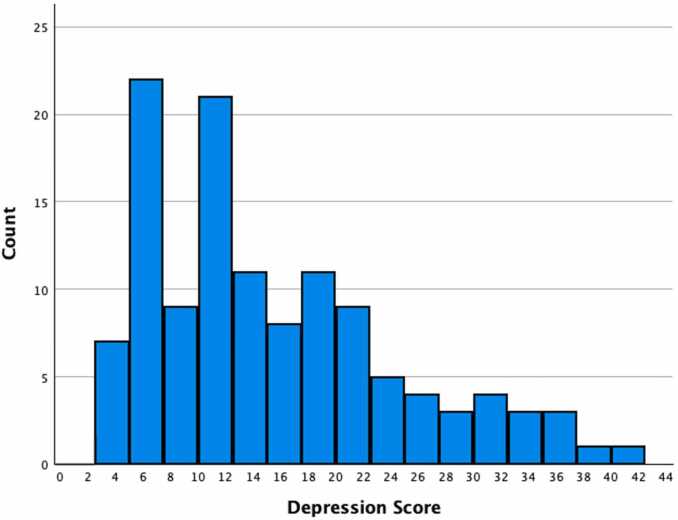
Prenatal Depression Scores. Distribution of total CES-D depression scores in the sample. In the present sample, depression scores ranged from 2 to 42 (median = 13.00). Thirty-nine percent of mothers endorsed clinical symptoms of depression.

Overall, CES-D total scores among the women in the current community sample ranged from 2 to 42. Thirty nine percent of mothers endorsed clinically elevated PMD symptoms (*n* = 48; see Fig. 1). The median CES-D total score of 13 was below the clinical cut off of 16 and reflects heightened, or at-risk levels of depression.

### 3.2 Data descriptives and covariates

The average number of fMRI frames for each participant was 237 (*SD* = 97, *min* = 106, *max* = 605). The maximum average rotation was 0.02 mm (*SD* =0.02, *min* = <0.01, *max* =0.09), while the maximum average linear movement was 0.41 mm (*SD* = 0.22, *min* = 0.08, *max* = 1.09). Sensitivity analyses addressing potential confounds are reported in the Supplemental Material.

### 3.3 Graph features related to PMD and age

**Fig. 2:**
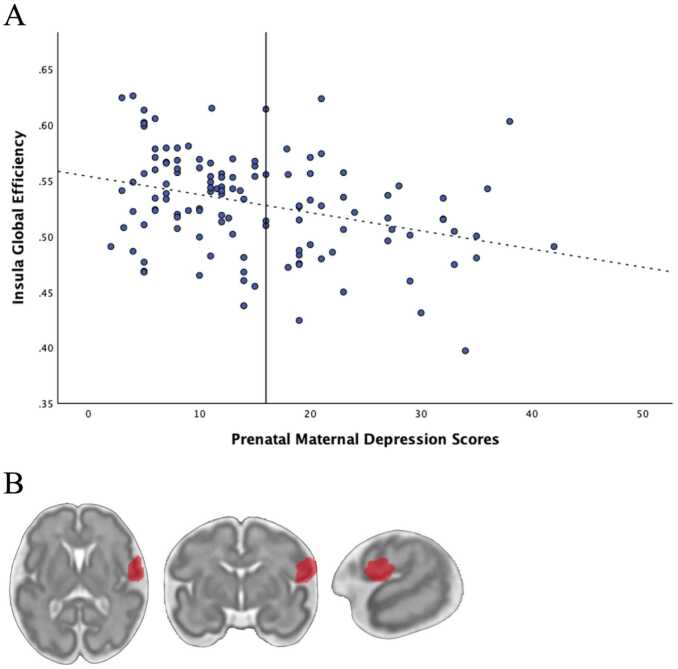
Prenatal Maternal Depression Symptoms and the Right Insula. **(A)** Scatterplot depicting Right Insula Global Efficiency and maternal depression symptom scores as reported on the CES-D. The vertical line at CES-D score 16 marks the clinical cutoff for the CES-D measure. **(B)** A visual of the brain region that had a significant reduction in global efficiency when examining the effect of PMD symptoms. This region has been classified as the right insula.

One parcel located in the right insular brain region had a significant negative association between global efficiency and PMD symptoms (*b* = −0.001, *t*(117) = -3.84, *p-FDR corrected* = .039) (see Fig. 2 and Table 3). Global efficiency of the right insula was extracted to derive growth plots (Fig. 2A). Alternative approaches exist that use categorical classifications of depression rather than continuous measure of symptoms. In our sample, classification of depression based on clinical cut off scores on the CES-D (i.e., scores > 16) were negatively correlated with insula global efficiency findings (*r* (121)= -.282, *p* = .002), such that depression was associated with reduced insula global efficiency.

**Table 3:** Associations between Maternal Prenatal Depression Symptoms and Brain Efficiency.

Region	Hemisphere	Direction	Peak coordinates			*b*	*p*
			*x*	*y*	*z*		
Global Efficiency							
Insula	Right	Negative	37	12	12	−0.00	< .001

With interest in associations that do not meet the stringent false discovery rate (FDR) correction, whole-brain results, reported at *p* < .05, *uncorrected* are reported in Supplemental Material Table 1.

**Fig. 3:**
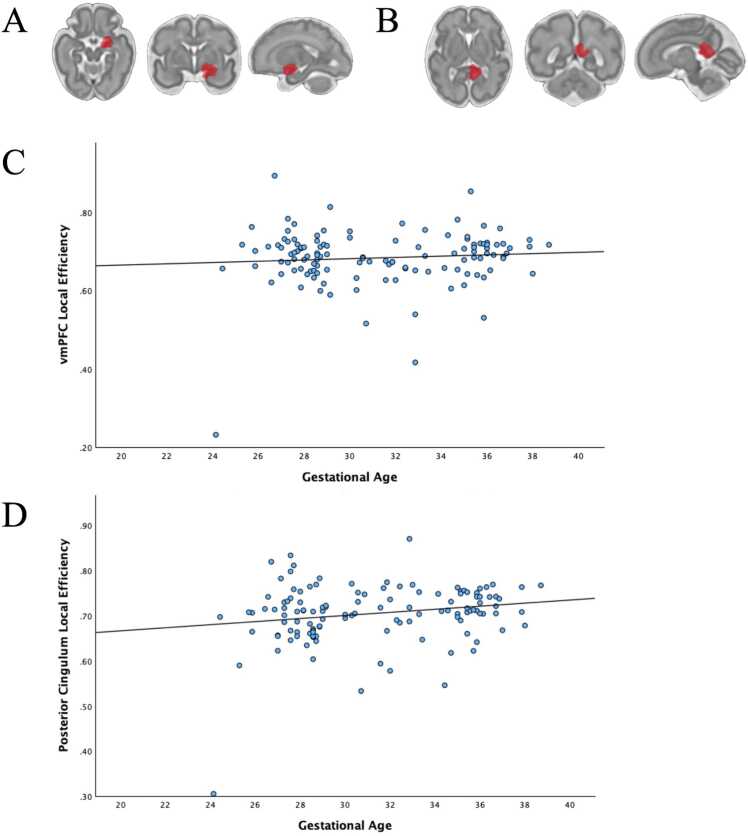
Fetal Age and Brain Regions that Demonstrated Increased Local Efficiency. When examining the effect of fetal age, the following brain regions had significant increases in lobal efficiency. **(A)** This region has been classified as the ventral medial prefrontal cortex. **(B)** This region has been classified as the right posterior cingulum. **(C)** Scatterplot depicting right vmPFC Local Efficiency and Gestational Age. **(D)** Scatterplot depicting right Posterior Cingulum Local Efficiency and Gestational Age.

Fetal age had modest associations with increased local efficiency in a parcel located in the right ventral medial prefrontal cortex (*b* = 0.01, *t*(120) = 3.68, *p-FDR corrected* = .041) and a parcel located in the right posterior cingulate cortex (*b* = 0.01, *t*(120) = 3.63, *p-FDR corrected* = .041) (see Fig. 3). Additional age effects are reported in Supplemental Material Table 2 at a reduced threshold of *p* < .05, *uncorrected*. Results of this additional exploratory analyses reveal that the majority of regions showing age effects demonstrate increased efficiency with advancing age.

## 4 Discussion

The current study addresses possible differences in local and global network efficiency that may underly known associations between PMD symptoms and child cognitive and affective problems. We observed a significant relationship between PMD and global efficiency in the right fetal insula (*p* FDR-corrected=.039). We did not observe PMD differences in local efficiency in any brain regions (*p* < 0.05, FDR-corrected). Global efficiency is a reflection of connectivity of a given region to all other nodes in the network, whereas local efficiency evaluates only the nearest neighboring nodes. Thus, the observed effect in the insula likely reflects contributions of the insula to the whole network architecture, rather than the local network. Fetal age-related increases in local efficiency in the right posterior cingulum and the ventral medial prefrontal cortex demonstrate strengthening connections with neighboring brain regions in these areas as opposed to widespread network communications. These are the first data to show intrauterine onset of PMD-related global insula architecture differences emerging before birth.

The observed difference in the offspring insula is interesting given that the insula is an important constituent of the human limbic system. More specifically, the insula is a contributor to the limbic system regions involved in emotions and communicates with the orbitofrontal cortex, amygdala, and the anterior cingulate cortex, while the insula is less involved in limbic system regions associated with memory and spatial functioning such as the hippocampus and connected structure (Rolls, 2015). It is possible that observing differences in this network, and specifically within the insula, before birth contributes to early emotion processing and regulation in the infant. PMD has been associated with children's social and emotional functioning throughout childhood and increased risk for developing a psychiatric disorder (Glover, 2011, Madigan et al., 2018). In the current analysis, we showed that global efficiency of the fetal insula is reduced in fetuses of mothers with a higher level of PMD symptoms.

Influence of differential global insula efficiency in the fetal brain may also be relevant to patterning of total network architecture. The insula is uniquely seated as a highly connected area of integration and is the hub to total network connectivity even in childhood (Oldham and Fornito, 2019). Interestingly, Arichi et al. (2017) have also shown that spontaneous waves of electrical activity that pattern brain organization at the beginning of life have origins in cortical regions that encompass the insula. It is possible that PMD-related insula differences are instructive to global maturational trajectories with implications for life-long health.

Having observed adjusted global efficiency in the insula brain region of fetuses whose mothers reported higher levels of PMD symptoms further exemplifies the importance of insular functional connectivity at the fetal stage of development. Indeed, efficiency in global connectivity generally relates to shorter path length between any two nodes in a network. Turk et al. (2019) examined fetal brain functioning at 20–40 weeks gestation and found that the insular and frontal lobes were the most densely connected regions in the fetal brain. The insula brain region is one of the first subcortical structures to mature and has detectible modularity in neonates (Afif et al., 2007; Alcauter et al., 2015a). When examining the current results using a life course perspective, the decreased efficiency of a highly connected brain region during the fetal period could have cascading effects on child neurocognitive development and, in part, explain the transgenerational risk for depression.

Our PMD related decrease in fetal insula global efficiency findings are inconsistent with infant and child studies, which have reported increased insular global efficiency. Donnici et al. (2023) found that children between ages 2.6–8 years of age who were exposed to higher PMD symptoms were slower to present with age related changes in limbic global efficiency and average node degree. Expected age related changes included: increased graph theory metrics (i.e., global and local efficiency among others) in the DMN, and both increased global efficiency and node degree in the limbic system, which includes the insula. Rotem-Kohavi et al., (2019) found that infants whose mothers endorsed greater PMD symptoms had higher connector hub values in the insula, left anterior cingulate, and caudate, and higher provincial hub values in the amygdala when functional connectivity was assessed via modularity and connector/provincial hubs collected during resting-state. Additionally, no differences in global or local efficiency have been found in neonates exposed to PMD (Wang et al., 2022). However, other measures of functional connectivity (i.e., strength of functional connectivity during rs-fNIRS) revealed decreased connectivity in the left frontal-parietal and temporal-parietal brain regions in neonates. Our sample consists of fetuses who display altered topological features from that of an infant or child, and future research should follow offspring brain functioning exposed to PMD longitudinally throughout development, starting in utero.

In order to understand the increased fetal insula global efficiency findings fully as it relates to PMD exposure, we must place these results in context of typical fetal brain FC in the third trimester of pregnancy. The gestational age of the fetuses included in this analysis ranged from 24.14 – 38.71 weeks. The current study did not find age related global efficiency effects in the insula. Therefore, we cannot conclude whether PMD is associated with either an accelerated or delayed developmental pattern (Di Martino et al., 2014). We did find a modest increase in local efficiency in the right vmPFC and anterior cingulum in older fetuses, which provides insight into the rapid functional development of the fetal brain. The vmPFC is a key developing connectivity hub in the fetal brain, and it becomes increasingly more integrated with its neighboring brain regions as the brain matures. The posterior cingulum also demonstrated this same pattern of increased local efficiency. Our age-related results are similar to existing articles that explore whole brain fetal development and have found positive associations between local efficiency and age, but no age related changes in global efficiency (De Asis-Cruz et al., 2021, Kim et al., 2023). Notably, dramatic neural, structural, and functional brain changes occur during the third trimester of pregnancy (Andersen, 2003; Turk et al., 2019).

The prenatal period presents a unique opportunity for examining intergenerational transfer of risk, because it removes the contribution of postnatal factors that are known to influence infant and child brain and behavioral development. For example, we know that early caregiving is an influential factor in child emotional and behavioral development associated with risk for later psychiatric disorders (Ahun et al., 2018, Gelaye et al., 2016). Studies have shown that maternal postnatal depression is associated with structural and functional brain development in infancy and throughout childhood (Luking et al., 2011, Thibodeau et al., 2006). Postnatal child brain development is commonly impacted by parenting behaviors and the home environment, while alterations to fetal brain FC are likely due to the in utero environment (Bock et al., 2015, Osborne et al., 2018, Sandman et al., 2015). The goal of the present study was achieved by providing evidence of altered connectivity in utero before the postnatal environment is able to exert its effects.

We were interested in the prenatal brain from a developmental life course perspective. There are similarities in how fetal and adult brains functionally communicate; however, significant changes occur throughout pregnancy, childhood, and at each stage in the life course, and yet, fetal brain FC lays a foundation that impacts the trajectory of functional brain development (Edde et al., 2021, Keunen et al., 2017, Turk et al., 2019). There are several proposed mechanisms for how exposure to PMD and atypical child brain structure and function are associated, including genetics, the in utero environment, and continued maternal depression throughout development. Elevated stress hormones, specifically cortisol, has been proposed as a mechanism for how PMD changes the fetal brain (Bock et al., 2015, Osborne et al., 2018, Sandman et al., 2015). Genetics plays a role in the risk for depression, and there is evidence of intergenerational transmission of risk from mothers to offspring (LeMoult et al., 2015). However, not all children of mothers with PMD go on to develop psychiatric disorders, indicating that environmental factors also play a role in the transmission of risk (LeMoult et al., 2015). The prenatal environment and, more specifically, changes made to the fetal brain in utero, may have cascading effects on developmental trajectories and put an individual at risk for experiencing depression later in life.

### 4.1 Strengths and limitations

A limitation of the current neuroscience research field is an overrepresentation of white middle-class study samples, whose data is not appropriately generalizable to low-income or ethnic-racial minority groups (Falk et al., 2013, Garcini et al., 2022, Heller et al., 2014, Nketia et al., 2021). The current study’s sample consisted of primarily low-income African American pregnant women who are at greater risk of experiencing prenatal depression and other health related disparities (Melville et al., 2010). Depression is also more common among low SES populations who often lack access to quality mental health care services (Goyal et al., 2010). The current analysis helps to fill this gap in literature by studying a primarily African American sample, but this reduces generalizability to other populations who are at a reduced risk of experiencing PMD.

The current analysis adds to the limited research on fetal fMRI and demonstrates how PMD is associated with changes in fetal functional connectivity. The exact pathway by which PMD influences child development must be better understood to provide effective and timely psychological intervention and contribute to the theoretical understanding of child development within the context of neurobiology. Studying the fetal brain eliminates the effects of the postnatal environment on child brain development, such as ongoing maternal psychiatric symptomatology and the impact it has on the mother-child relationship (Slomian et al., 2019). However, there are additional difficulties when studying fetal fMRI in utero which has limited research capabilities to examine the mechanisms for how this association between PMD and child outcomes occurs, and the exact role fetal functional MRI plays (Rajagopalan et al., 2021, Van den Heuvel and Thomason, 2016). One of the greatest challenges in fetal fMRI analysis is high and complex motion (Van den Heuvel and Thomason, 2016). It will be valuable for future work to evaluate the optimal inclusion/exclusion criteria for motion censoring thresholds and low motion frame count retention in fetal humans to assess the ideal balance between maximizing quantity of data while optimizing data quality, and to do so across datasets with variable thresholds.

Examining PMD alone is examining a small scope of psychiatric symptoms that influence the intrauterine environment and could alter fetal brain functioning and development. The current analysis focused on PMD symptoms, given that depression is the primary psychiatric disorder affecting soon-to-be mothers (Van Niel and Payne, 2020). However, depression is a narrow construct. Prior literature has examined prenatal stress, anxiety, traumatic events, and other relevant influences on the mother that, in turn, would change the fetal environment, but did not assess fMRI using graph theory in a fetal sample (Graignic-Philippe et al., 2014, Van den Bergh et al., 2018). An interesting direction for future work would be to examine alternative psychiatric symptoms and traumatic events to establish whether these have variant effects on fetal functional brain development.

Many factors likely play a role in any study that looks at the association between maternal mental health and offspring brain development. Examples of these factors are prenatal social support, maternal coping and self-regulation abilities, or biological measures of maternal stress among others. These factors could impact fetal functional connectivity when studying the context of PMD. Future research studies should examine these factors to deepen our understanding of the prenatal environment and fetal brain FC (Graignic-Philippe et al., 2014). Large scale studies such as Healthy Brain and Child Development (HBCD) and Adolescent Brain Cognitive Development (ABCD) would be better suited to address these inquiries because the scope of these questions require large samples (Casey et al., 2018, Nelson et al., 2024).

## 5 Conclusion

The current study examines possible fetal origins to explain the observed association between PMD and increased offspring risk for depression, including altered functional brain connectivity. This is a critical topic because perinatal depression is highly prevalent (Van Niel and Payne, 2020) and yet there is limited data isolating intrauterine brain effects that may underlie the intergenerational risk for depression. Our graph theory analysis revealed a significant association between increased PMD symptoms and decreased global efficiency in the right insular brain region. Contrary to our hypothesis, there were no significant associations between maternal prenatal depressive symptoms and reduced global or local efficiency in any other brain networks. However, when the statistical threshold was reduced and FDR was uncorrected, several associations were significant at a *p* < .05 level. These mixed findings highlight the need to study the developing brain in utero. These results call for early intervention to better support soon-to-be mothers at risk for experiencing depression, and particularly those at greater risk of experiencing the real-world repercussions of depression such as limited access to resources, reduced social support, increased psychosocial stress, and poor relationships with health care providers, to name a few. These results also contribute to the broader research on fetal brain functioning and put these findings within context of PMD to better understand what constitutes risk.

## CRediT authorship contribution statement

**Mark Duffy:** Writing – review & editing, Data curation. **Iris Menu:** Data curation. **Christopher Trentacosta:** Writing – review & editing, Investigation, Funding acquisition, Conceptualization. **Thomason Moriah E:** Writing – original draft, Visualization, Supervision, Investigation, Funding acquisition, Conceptualization. **Reed Ellyn C:** Writing – review & editing, Writing – original draft, Visualization, Validation, Project administration, Methodology, Investigation, Formal analysis, Data curation, Conceptualization. **Lanxin Ji:** Writing – original draft, Software, Methodology, Funding acquisition, Formal analysis. **Marjorie Beeghly:** Writing – review & editing, Conceptualization. **Amyn Majbri:** Methodology, Data curation. **Tanya Bhatia:** Data curation.

## Declaration of Competing Interest

The authors declare that they have no known competing financial interests or personal relationships that could have appeared to influence the work reported in this paper.

The author is an Editorial Board Member/Editor-in-Chief/Associate Editor/Guest Editor for *[Journal name]* and was not involved in the editorial review or the decision to publish this article.

## Data Availability

Data from the fetal neuroimaging cohort these data are drawn from are available on OpenNeuro and on the NIMH NDAR Data Archive. Codes for processing fetal fMRI data are available on Zenodo.

## Footnotes

Appendix A Supplementary data associated with this article can be found in the online version at doi:10.1016/j.dcn.2025.101585.
