# Supplementary material for: Fetal functional connectivity: Examining the role of prenatal maternal depression symptoms using graph theory
Source: Dev Cogn Neurosci. 2025 Jun 16;74:101585. doi: 10.1016/j.dcn.2025.101585 (PMC12221684; doi:10.1016/j.dcn.2025.101585)
Supplement: Supplementary file 1 — Supplementary material [file mmc1.docx]

**Supplemental Material**

**Fetal functional connectivity: Examining the role of prenatal maternal depression symptoms using graph theory**

Ellyn Reed, Lanxin Ji, Marjorie Beeghly, Amyn Majbri, Tanya Bhatia, Mark Duffy, Iris Menu, Christopher Trentacosta_,_ Moriah E. Thomason.

**Whole-brain PMD and graph efficiency at *p* < 0.05, uncorrected.** Rigor of the present study required FDR correction across brain regions examined. For PMD, the area that survived FDR was the right insula. This region is a connectivity hub even early in life (Gao et al., 2011; Thomason, 2018), and a key node in the Salience Network (Seeley, 2019). Reduced efficiency before birth may have cascading effects given the early wide-scale connectivity of this region. Our expanded analysis explored the whole brain without applying FDR and reports this as supplemental information in Table 1, below. This expanded analysis shows wide-scale efficiency differences related to PMD, with mixed directionality. Strong positive effects between PMD and global efficiency are seen in the cingulum and middle occipital regions (*p’s* < 0.008). These regions are of interest as they are early developing brain regions that, like the insula, are reported as highly connected brain regions even in earliest examinations of functional connectivity. Van den Heuvel and colleagues report primary visual cortex as a hub in the fetal brain in their 2018 paper (van den Heuvel et al., 2018). Thomason and colleagues observed increased negative cingulate connectivity with advancing fetal age, noting that this region will also have many negative functional connections in the mature adult form (Thomason et al., 2014). It is interesting that the regions showing the strongest associations with PMD are all early-emerging connectional hubs. This highlights potential significance of PMD-related differences to total brain network architecture.

| **Supplemental Table 1.**   \| *Associations between Maternal Prenatal Depression Symptoms and Brain Efficiency* \| \| \| \| \| \| \| \| \| \| --- \| --- \| --- \| --- \| --- \| --- \| --- \| --- \| --- \| \| Region \| Hemisphere \| Direction \| Peak coordinates \| \| \|  \| *b* \| *p* \| \|  \|  \|  \| *x* \| *y* \| *z* \|  \|  \|  \| \| Global Efficiency \|  \|  \|  \|  \|  \|  \|  \|  \| \| Posterior Cingulum \| Left \| Positive \| -3 \| -10 \| 11 \|  \| 0.00 \| 0.006 \| \| Middle Occipital \| Left \| Positive \| -25 \| -29 \| 10 \|  \| 0.00 \| 0.008 \| \| Posterior Cuneus \| Left \| Positive \| -3 \| -40 \| 1 \|  \| 0.00 \| 0.011 \| \| Insula \| Right \| Negative \| 37 \| 12 \| 12 \|  \| -0.00 \| <.001* \| \| Operculum \| Left \| Negative \| -22 \| 16 \| -2 \|  \| -0.00 \| 0.020 \| \| Preuncus \| Right & Left \| Negative \| 3 \| -16 \| 19 \|  \| -0.00 \| 0.030 \| \| Cerebellum \| Right & Left \| Negative \| -9 \| -23 \| -18 \|  \| -0.00 \| 0.034 \| \| Inferior frontal \| Left \| Negative \| -28 \| 32 \| 11 \|  \| -0.00 \| 0.038 \| \| Inferior frontal \| Left \| Negative \| -25 \| 36 \| 0 \|  \| -0.00 \| 0.041 \| \| Middle Cingulate \| Right & Left \| Negative \| 3 \| 13 \| 20 \|  \| -0.00 \| 0.049 \| \| Posterior Insula \| Left \| Negative \| -22 \| 5 \| 5 \|  \| -0.00 \| 0.049 \| \| Local Efficiency \|  \|  \|  \|  \|  \|  \|  \|  \| \| Supplementary Motor \| Right \| Positive \| 6 \| 22 \| 25 \|  \| 0.00 \| 0.022 \| \| Postcentral \| Left \| Positive \| -33 \| -1 \| 23 \|  \| 0.00 \| 0.022 \| \| Anterior Middle Temporal Pole \| Left \| Positive \| -21 \| 18 \| -15 \|  \| 0.00 \| 0.035 \| \| Thalamus \| Right \| Positive \| 7 \| 5 \| 7 \|  \| 0.00 \| 0.038 \| \| Temporal \| Right \| Positive \| 24 \| 9 \| -20 \|  \| 0.00 \| 0.038 \| \| Middle Cingulum \| Right & Left \| Positive \| 3 \| -2 \| 19 \|  \| 0.00 \| 0.040 \| \| Inferior Parietal \| Left \| Positive \| -30 \| -15 \| 24 \|  \| 0.00 \| 0.043 \| \| Posterior Insula \| Left \| Negative \| -22 \| 5 \| 5 \|  \| -0.00 \| 0.020 \| \| Anterior Fusiform \| Right \| Negative \| 25 \| -2 \| -15 \|  \| -0.00 \| 0.030 \| \| Anterior Operculum \| Left \| Negative \| -28 \| 20 \| 12 \|  \| -0.00 \| 0.025 \| \| Motor \| Right \| Negative \| 26 \| 6 \| 25 \|  \| -0.00 \| 0.047 \| \| *p* values are reported with the reduced threshold of FDR uncorrected. The * indicates that FDR was corrected for. \| \| \| \| \| \| \| \| \|   **Whole-brain PMD and graph efficiency at *p* < 0.05, uncorrected.** Primary age effects are reported in the manuscript at *p* <0.05 FDR corrected. Significant age effects were observed ventral medial prefrontal cortex (vmPFC) (*b* = 0.01, *t*(120)= 3.68, *p-FDR corrected* = .041) and right posterior cingulate cortex (PCC). These regions showed increased *local* efficiency with age. Uncorrected age effects for all brain areas are reported below as exploratory supplemental analyses at *p* < 0.05, uncorrected. We observe the strongest age to *global* efficiency effect in the medial visual region (*p-FDR corrected* = .056). As discussed above, the visual cortex has been identified as a hub in fetal brain development (van den Heuvel, 2018), suggesting global centrality of this region in early human life. Here, we show that global efficiency of this region increases in fetal life. A recent study by Ji and colleagues examined connectivity of the visual cortex over the birth transition. They show strong increased connectivity within visual systems at birth, along with reduced connectivity of visual systems to temporal regions following birth (Ji et al., 2024). Thus, the maturational trajectory of the fetal visual cortex may be overgrowth in fetal life (increased global efficiency with fetal age) followed by decreased connectivity to non-visual areas after birth.  **Supplemental Table 2.**  *Associations between Fetal Age and Brain Efficiency*   \| Region \| Hemisphere \| Direction \| Peak coordinates \| \| \|  \| *b* \| *p-unc* \| *p-FDR* \| \| --- \| --- \| --- \| --- \| --- \| --- \| --- \| --- \| --- \| --- \| \|  \|  \|  \| *x* \| *y* \| *z* \|  \|  \|  \|  \| \| Global Efficiency \|  \|  \|  \|  \|  \|  \|  \|  \|  \| \| Medial Visual \| Right \| Positive \| 16 \| -17 \| 2 \|  \| 0.00 \| <0.001 \| 0.056 \| \| Operculum \| Right \| Positive \| 29 \| -2 \| 12 \|  \| 0.00 \| 0.002 \| 0.109 \| \| Lingual \| Left \| Positive \| -11 \| -19 \| 4 \|  \| 0.00 \| 0.002 \| 0.109 \| \| Medial Visual \| Left \| Positive \| -4 \| -29 \| 1 \|  \| 0.00 \| 0.002 \| 0.109 \| \| Medial Visual \| Right \| Positive \| 11 \| -23 \| 11 \|  \| 0.00 \| 0.002 \| 0.109 \| \| Orbital Medial Frontal \| Left & Right \| Positive \| 1 \| 40 \| -1 \|  \| 0.00 \| 0.004 \| 0.116 \| \| Posterior Cuneus \| Left \| Positive \| -3 \| -40 \| 1 \|  \| 0.00 \| 0.004 \| 0.116 \| \| Putamen \| Left \| Positive \| -12 \| 12 \| 5 \|  \| 0.00 \| 0.005 \| 0.116 \| \| Thalamus \| Right \| Positive \| 15 \| -2 \| 5 \|  \| 0.00 \| 0.005 \| 0.116 \| \| Frontal Inferior Orbital \| Right \| Negative \| 27 \| 35 \| -3 \|  \| -0.00 \| 0.006 \| 0.999 \| \| Local Efficiency \|  \|  \|  \|  \|  \|  \|  \|  \|  \| \| Ventral Medial Prefrontal \| Right \| Positive \| 14 \| 12 \| -7 \|  \| 0.01 \| <0.001 \| 0.021* \| \| Posterior Cingulum \| Right \| Positive \| 7 \| -11 \| 10 \|  \| 0.01 \| <0.001 \| 0.021* \| \| Anterior Cingulum \| Right \| Positive \| 2 \| 27 \| -0 \|  \| 0.01 \| 0.004 \| 0.289 \| \| Paracentral Lobule \| Left & Right \| Negative \| 3 \| 2 \| 35 \|  \| -0.00 \| 0.004 \| 0.349 \| \| Motor \| Right \| Negative \| 27 \| 20 \| 27 \|  \| -0.00 \| 0.005 \| 0.349 \| \| Frontal Superior \| Left \| Negative \| -5 \| 49 \| 4 \|  \| -0.00 \| 0.008 \| 0.349 \| \| Ventral Medial Prefrontal \| Right \| Negative \| 6 \| 33 \| -11 \|  \| -0.00 \| 0.008 \| 0.349 \| |
| --- | --- | --- | --- | --- | --- | --- | --- | --- | --- | --- | --- | --- | --- | --- | --- | --- | --- | --- | --- | --- | --- | --- | --- | --- | --- | --- | --- | --- | --- | --- | --- | --- | --- | --- | --- | --- | --- | --- | --- | --- | --- | --- | --- | --- | --- | --- | --- | --- | --- | --- | --- | --- | --- | --- | --- | --- | --- | --- | --- | --- | --- | --- | --- | --- | --- | --- | --- | --- | --- | --- | --- | --- | --- | --- | --- | --- | --- | --- | --- | --- | --- | --- | --- | --- | --- | --- | --- | --- | --- | --- | --- | --- | --- | --- | --- | --- | --- | --- | --- | --- | --- | --- | --- | --- | --- | --- | --- | --- | --- | --- | --- | --- | --- | --- | --- | --- | --- | --- | --- | --- | --- | --- | --- | --- | --- | --- | --- | --- | --- | --- | --- | --- | --- | --- | --- | --- | --- | --- | --- | --- | --- | --- | --- | --- | --- | --- | --- | --- | --- | --- | --- | --- | --- | --- | --- | --- | --- | --- | --- | --- | --- | --- | --- | --- | --- | --- | --- | --- | --- | --- | --- | --- | --- | --- | --- | --- | --- | --- | --- | --- | --- | --- | --- | --- | --- | --- | --- | --- | --- | --- | --- | --- | --- | --- | --- | --- | --- | --- | --- | --- | --- | --- | --- | --- | --- | --- | --- | --- | --- | --- | --- | --- | --- | --- | --- | --- | --- | --- | --- | --- | --- | --- | --- | --- | --- | --- | --- | --- | --- | --- | --- | --- | --- | --- | --- | --- | --- | --- | --- | --- | --- | --- | --- | --- | --- | --- | --- | --- | --- | --- | --- | --- | --- | --- | --- | --- | --- | --- | --- | --- | --- | --- | --- | --- | --- | --- | --- | --- | --- | --- | --- | --- | --- | --- | --- | --- | --- | --- | --- | --- | --- | --- | --- | --- | --- | --- | --- | --- | --- | --- | --- | --- | --- | --- | --- | --- | --- | --- | --- | --- | --- | --- | --- | --- | --- | --- | --- | --- | --- | --- | --- | --- | --- | --- | --- | --- | --- | --- | --- | --- | --- | --- | --- | --- | --- | --- | --- | --- | --- | --- | --- | --- | --- | --- | --- | --- | --- | --- | --- | --- | --- | --- | --- | --- | --- | --- | --- | --- | --- | --- | --- | --- | --- | --- | --- | --- | --- | --- | --- | --- | --- | --- | --- | --- | --- | --- | --- | --- | --- | --- | --- | --- | --- | --- | --- | --- | --- | --- | --- | --- | --- | --- | --- | --- | --- | --- | --- | --- | --- | --- | --- | --- | --- | --- | --- | --- | --- | --- | --- | --- | --- | --- | --- | --- | --- | --- | --- | --- | --- | --- | --- | --- | --- | --- | --- | --- | --- | --- | --- | --- | --- | --- | --- | --- | --- | --- | --- | --- | --- | --- | --- | --- | --- | --- | --- | --- | --- | --- | --- | --- | --- | --- | --- | --- | --- | --- | --- | --- | --- | --- | --- | --- | --- | --- | --- | --- | --- | --- | --- | --- | --- | --- |

*p* values are reported with the reduced threshold of FDR uncorrected and after FDR was corrected for. The * indicates that finding was significant when FDR was corrected for.

**Sensitivity Analysis.** We examined relationships of multiple potentially influential variables with PMD, our independent variable, specifically mental and physical health factors. For those that were significant we went on to test potential influence on our insula finding.

Prenatal stress was measured using the Perceived Stress Scale (PSS; Cohen et al., 1983). Prenatal anxiety was measured using the State Trait Anxiety Inventory (STAI; Spielberger et al., 1970). Prenatal substance use was measured both from self-report and medical record review. The self-report measure was a health behaviors questionnaire (adapted from Jackson, 2006) that contains a substance use subscale. Higher scores on the health behavior substance use subscale reflect lower use of alcohol and/or tobacco. A subset of 80 participants had urine test results during pregnancy measuring amphetamines, barbiturates, benzodiazepines, cannabis, cocaine, methadone, or opiates. A total of 31 participants screened positive for any drug (barbiturates *N* = 1, benzodiazepines *N* = 2, cannabis *N* = 29, cocaine *N* = 1, methadone *N* = 1, or opiates *N* = 4).

Pearson correlation analyses determined the association between PMD and PSS, STAI, self-reported substance use, and other health related behaviors. A Bonferroni correction was used to compute a corrected threshold for significance (*p* < .0017). There was a significant positive correlation between PMD and both PSS and STAI at significance level of *p* < .0017, such that higher PMD symptoms was associated with increased prenatal stress and anxiety. There was a significant negative correlation between PMD and the healthy behaviors total score, substance abuse subscale, and sleep subscale at a significance level of *p* < .0017, such that higher PMD was associated with lower total healthy behaviors, increased alcohol and/or tobacco use, and decreased sleep. PMD was not significantly correlated with the healthy behaviors diet subscale, medical adherence subscale, nor the exercise subscale (*p* > .0017). An independent samples t-test revealed no significant difference in PMD scores between subjects who screened positive for any drug use during pregnancy via medical records and subjects who did not screen positive for any drug use (*p* > .0017).

Separate partial correlation analyses were conducted to determine the association between the fetal insula global efficiency results and PSS, STAI, total health behaviors, the healthy behavior sleep subscale, and the healthy behavior substance abuse subscale while controlling for the same covariates that were selected *a priori* (fetal gestational age, fetal sex, maternal age, and maternal education). Results revealed that the fetal insula global efficiency finding was not significantly correlated with STAI, PSS, total health behaviors, the healthy behavior sleep subscale, or the healthy behavior substance abuse subscale (*p* > .0017).

**References**

Cohen, S., Kamarck, T., & Mermelstein, R. (1983). A global measure of perceived stress. *Journal of health and social behavior*, 385-396. <https://doi.org/10.2307/2136404>

Gao, W., Gilmore, J. H., Giovanello, K. S., Smith, J. K., Shen, D., Zhu, H., & Lin, W. (2011). Temporal and spatial evolution of brain network topology during the first two years of life. *PloS one*, *6*(9), e25278. DOI: [10.1371/journal.pone.0025278](https://doi.org/10.1371/journal.pone.0025278)

Jackson, T. (2006). Relationships between perceived close social support and health practices within community samples of American women and men. *The Journal of psychology*, *140*(3), 229-246.

Ji, L., Menu, I., Majbri, A., Bhatia, T., Trentacosta, C. J., & Thomason, M. E. (2024). Trajectories of human brain functional connectome maturation across the birth transition. *PLoS Biology*, *22*(11), e3002909. DOI: [10.1371/journal.pbio.3002909](https://doi.org/10.1371/journal.pbio.3002909)

Seeley, W. W. (2019). The salience network: a neural system for perceiving and responding to homeostatic demands. *Journal of Neuroscience*, *39*(50), 9878-9882. DOI: [10.1523/JNEUROSCI.1138-17.2019](https://doi.org/10.1523/JNEUROSCI.1138-17.2019)

Spielberger, C. D., Gorsuch, R. L., & Lushene, R. E. (1970). Manual for the state-trait anxiety inventory.

Thomason, M. E., Brown, J. A., Dassanayake, M. T., Shastri, R., Marusak, H. A., Hernandez-Andrade, E., ... & Romero, R. (2014). Intrinsic functional brain architecture derived from graph theoretical analysis in the human fetus. *PLoS one*, *9*(5), e94423.

Thomason, M. E. (2018). Structured spontaneity: building circuits in the human prenatal brain. *Trends in neurosciences*, *41*(1), 1-3. DOI: [10.1016/j.tins.2017.11.004](https://doi.org/10.1016/j.tins.2017.11.004)

van den Heuvel, M. I., Turk, E., Manning, J. H., Hect, J., Hernandez-Andrade, E., Hassan, S. S., ... & Thomason, M. E. (2018). Hubs in the human fetal brain network. *Developmental cognitive neuroscience*, *30*, 108-115. DOI: [10.1016/j.dcn.2018.02.001](https://doi.org/10.1016/j.dcn.2018.02.001)
